# A membrane-embedded pathway delivers general anesthetics to two interacting binding sites in the *Gloeobacter violaceus* ion channel

**DOI:** 10.1074/jbc.M117.780197

**Published:** 2017-04-18

**Authors:** Mark J. Arcario, Christopher G. Mayne, Emad Tajkhorshid

**Affiliations:** From the ‡ Center for Biophysics and Quantitative Biology,; § Department of Biochemistry, College of Medicine, and; ¶ Beckman Institute for Advanced Science and Technology, University of Illinois at Urbana-Champaign, Urbana, Illinois 61801

**Keywords:** anesthetic, computational biology, ion channel, membrane protein, molecular dynamics

## Abstract

General anesthetics exert their effects on the central nervous system by acting on ion channels, most notably pentameric ligand-gated ion channels. Although numerous studies have focused on pentameric ligand-gated ion channels, the details of anesthetic binding and channel modulation are still debated. A better understanding of the anesthetic mechanism of action is necessary for the development of safer and more efficacious drugs. Herein, we present a computational study identifying two anesthetic binding sites in the transmembrane domain of the *Gloeobacter violaceus* ligand-gated ion channel (GLIC) channel, characterize the putative binding pathway, and observe structural changes associated with channel function. Molecular simulations of desflurane reveal a binding pathway to GLIC via a membrane-embedded tunnel using an intrasubunit protein lumen as the conduit, an observation that explains the Meyer-Overton hypothesis, or why the lipophilicity of an anesthetic and its potency are generally proportional. Moreover, employing high concentrations of ligand led to the identification of a second transmembrane site (TM2) that inhibits dissociation of anesthetic from the TM1 site and is consistent with the high concentrations of anesthetics required to achieve clinical effects. Finally, asymmetric binding patterns of anesthetic to the channel were found to promote an iris-like conformational change that constricts and dehydrates the ion pore, creating a 13.5 kcal/mol barrier to ion translocation. Together with previous studies, the simulations presented herein demonstrate a novel anesthetic binding site in GLIC that is accessed through a membrane-embedded tunnel and interacts with a previously known site, resulting in conformational changes that produce a non-conductive state of the channel.

## Introduction

The discovery of small molecules, such as diethyl ether or chloroform, that cause reversible immobilization and loss of consciousness helped usher in the era of modern medicine and surgical therapy. Despite well over a century of research, however, an atomic-detailed mechanism of general anesthesia is still vague and incomplete. Although it was once generally accepted that anesthetics worked through a nonspecific membrane disruption mechanism (1, 2), that theory has become obsolete, and it is now known that most, if not all, anesthetics specifically target ion channels in the nervous system, most notably the Cys-loop family of pentameric ligand-gated ion channels (pLGIC)^2^ (3–6). This family of proteins, which acts in response to release of neurotransmitters from the presynaptic terminal, was the target for multiple pharmaceutical agents, including alcohol, barbiturates, and benzodiazepines, in addition to anesthetics (5, 7–11). Anesthetics are known to inhibit the excitatory, cation-permeable channels of this family, such as the serotonin and nicotinic acetylcholine receptors and potentiate the inhibitory, anion permeable channels, such as the glycine and γ-aminobutyric acid type A receptors (5, 8–11). The structural mechanism of channel modulation, however, is still poorly understood due to the challenge of resolving high-resolution structures of these eukaryotic receptors. Bacterial homologues to this family have been discovered (12), however, that are sensitive to clinical concentrations of anesthetics (13, 14). Crystal structures of these bacterial homologues, such as GLIC (*Gloeobacter violaceus* ligand-gated ion channel) (15–20) and ELIC (*Erwinia chrysanthemi* ligand-gated ion channel) (21, 22), have been solved both in the presence and absence of anesthetics and provide a tremendous opportunity to study the molecular mechanism behind pLGIC function and modulation.

**Figure 1.**
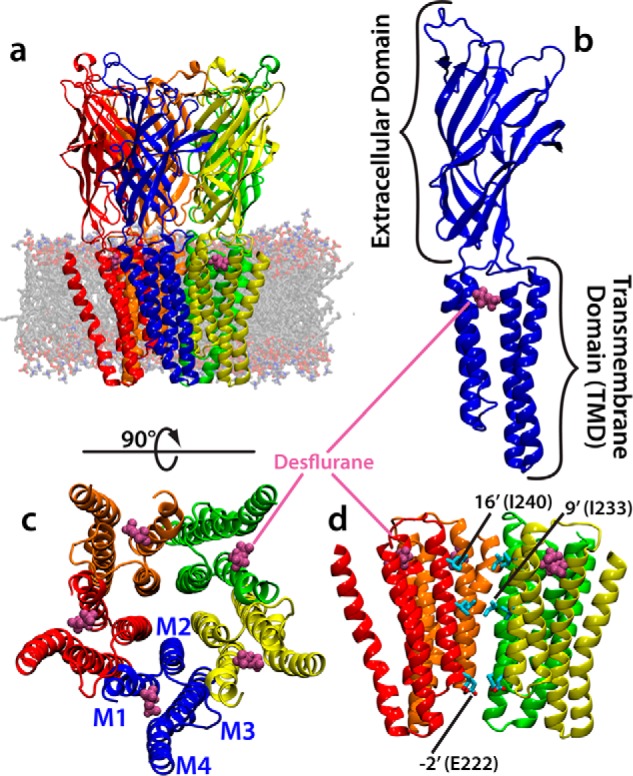
**Structure of GLIC.** *a*, molecular image of the equilibrated system containing GLIC embedded in a membrane. GLIC is shown in a multicolor representation, each subunit a different color, with the position of bound desflurane molecules shown in *magenta. b*, a single subunit of the homopentameric GLIC channel composed of an extracellular domain and transmembrane domain. This view is from outside the channel toward the ion conduction pore. The position of bound desflurane within the subunit is shown. *c*, top view of the transmembrane domain of GLIC. Each subunit is composed of four helices labeled *M1–M4* (shown in figure). The position of bound anesthetic is shown. *d*, side view of the ion conduction channel excluding one subunit for clarity. The position of −2′, 9′, and 16′ are shown for clarity. The hydrophobic gate is composed of the area between 9′ and 16′, whereas the selectivity filter is composed of the −2′ position.

GLIC is a proton-activated (pH_50_ ∼ 4.5–5.0), cation-permeable homologue of the Cys-loop receptor family (12, 23). The full bacterial channel is a homopentamer with a 5-fold symmetry axis aligned to the central ion conduction pore (Fig. 1). Each monomer is composed of an extracellular domain and a transmembrane domain (TMD) and notably lacks the intracellular domain found in eukaryotic pLGICs (3–6). The TMD of each subunit consists of four membrane-spanning helices, named M1-M4, with the M2 helix oriented toward the center of the channel to form the central pore in the assembled pentamer. It is thought that the 9′ and 16′ positions on the M2 helix in Cys-loop receptors (corresponding to Ile-233 and Ile-240 in GLIC) form the hydrophobic gate, which when the channel is closed, dehydrates the pore and prevents ion conduction (24–26). Moreover, a ring of glutamates at the intracellular mouth of the pore (−2′ or Glu-222) is known to play a significant role in ion translocation (27, 28) as well as act as a component of the selectivity filter for the channel (29).

Although multiple techniques have been used, there is little consensus regarding which binding site or sites are responsible for the observed clinical effects of anesthetics. The current debate is centered on two distinct binding sites named the intrasubunit (17) and intersubunit sites (30), with evidence supporting each site (6). Additionally, a pore-blocking site has been considered as an alternative hypothesis (31). Due to the multitude of general anesthetic binding sites identified, it has been proposed that multisite binding could be responsible for channel modulation (14, 32). Moreover, the open and closed states of pLGICs are poorly defined, and there is significant debate over whether the crystal structures reflect physiologically open or closed states, further convoluting an understanding of anesthetic action on these channels. Crystal structures of the “open” state of GLIC and “closed” state of ELIC (*E. chrysanthemi* ligand-gated ion channel) have been solved (15, 21, 33); however, neither the addition of anesthetics (17) nor the incorporation of mutations that constitutively activate these channels (22) demonstrate any significant change in protein structure.

The solution of atomic-resolution (2.9–3.1 Å) GLIC structures with bound anesthetics, however, presents an unprecedented opportunity to study how these molecules bind to and affect ion channel dynamics in atomic detail using molecular dynamics (MD) simulations (25, 31, 32, 34–37). Herein, a series of computational studies aimed at identifying the binding mechanism of the inhaled anesthetic, desflurane, and characterizing the conformational changes associated with anesthetic binding are presented. Multiple MD techniques were used to probe the interaction between desflurane and GLIC, including flooding, mutagenesis, and free energy simulations. These simulations revealed that desflurane enters and exits the protein via a membrane-embedded pathway, a previously uncharacterized phenomenon, and a result that helps to explain why anesthetic potency is closely related to hydrophobicity. Moreover, upon occupying the binding region, desflurane is held loosely in the binding pocket and is blocked from exiting the transmembrane lumen of GLIC by only a small grouping of bulky residues. Asymmetric occupation of the five binding regions leads to a detectable conformational change in the M2 helix that causes pore contraction and dehydration.

## Results and discussion

Mounting evidence shows that general anesthetics exert their effects via interaction with pLGICs (5, 7, 10, 11). Although earlier theories on anesthetic action proposed a nonspecific membrane disruption mechanism, recent simulations (38) demonstrate that concentrations of general anesthetic similar to those used in this study have no effect on the physical and chemical properties of a POPC (1-palmitoyl-2-oleyl-*sn*-glycero-3-phosphocholine) membrane. Moreover, experimental studies (39, 40) find that clinical concentrations of general anesthetics have no appreciable effect on lipid bilayers, whereas supraclinical concentrations cause slight perturbations in membrane structure. Here, multiple extended simulations were performed for membrane-embedded GLIC, including flooding and energetics calculations, to better understand how anesthetics bind to and affect pLGIC dynamics. Together, these simulations provide evidence that binding of general anesthetics to GLIC proceeds via a membrane-embedded pathway. Moreover, multiple anesthetics are needed to bind each subunit to exert their effects with establishment of an asymmetric ligand occupancy in GLIC leading to conformational changes that result in a non-conductive state.

### Desflurane reaches crystal structure binding site via a membrane-embedded pathway

**Figure 2.**
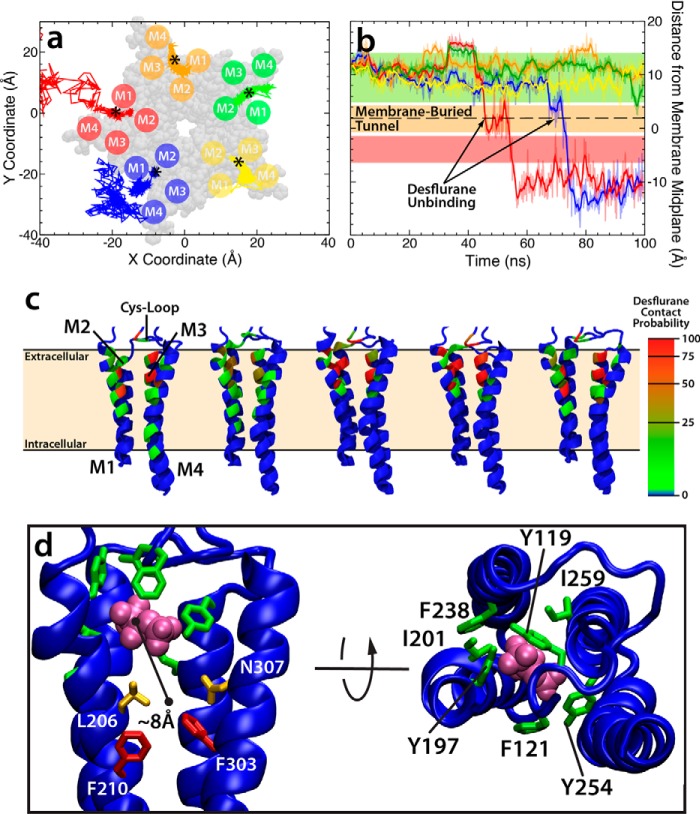
**Desflurane is loosely bound to the intrasubunit site identified in crystal structure.** The data in this figure comes from the 100-ns simulation starting from the fully bound state in which desflurane is initially bound as observed from the crystal structure. *a*, plot of the position of each anesthetic projected onto the *xy* plane as a function of time. In the case of the *blue* and *red* subunits, desflurane spontaneously and completely exits the protein. The *asterisk* denotes the initial center of mass of each anesthetic at *t* = 0 ns. The structure of the TMD is shown in *transparent gray* van der Waals representation. The approximate location of the M1-M4 helices in each subunit is shown as a *circle. b*, plot of the height of the center of mass of each anesthetic as a function of time. The *colored lines* here represent the subunit the anesthetic was initially bound to in *a*. The *colored bars* correspond to the position of the amino acids pictured in *d*. Desflurane contact probabilities (*c*) are mapped onto the backbone of each residue for individual subunits as discussed under “Experimental Procedures.” The color on the protein corresponds to how often the desflurane in that subunit contacts the protein (defined as within 3.5 Å of any atom in the residue) and is adjusted so that a contact probability >50% is *red*. The first two subunits from the *left* are those in which the desflurane unbinds and exits. *d*, molecular images showing side and top views of the position of bulky residues that block the exit (*green* and *red*) and residues with comparatively smaller side chains that allow desflurane to exit into the lipid bilayer (*orange*). The color of the residues in this image corresponds to the same *colored horizontal bars* in *b*.

Equilibrium simulation of anesthetic-bound GLIC (described in detail under “Experimental Procedures”), in which the position of the desflurane ligand was taken directly from the crystal structure (Fig. 1) (17), shows that desflurane molecules are only loosely associated with the protein (Fig. 2*a*). During the course of this simulation, each ligand explored, on average, 116 Å^3^ of space within the lumen formed by helices M1-M4. In fact, the binding arrangement observed from the crystal structure (17) shows no specific side-chain-anesthetic interactions to stabilize the anesthetic within this volume but, rather, provides a generally hydrophobic environment. Contact probabilities computed to quantify the extent of these diffuse interactions largely show even sampling of each residue in the binding region by the anesthetic (Fig. 2*c*, Table 1), supporting the idea that GLIC lacks a tight binding site that is specific to anesthetics. The volume explored by the anesthetic combined with an even sampling of residues that line the lumen of the binding region suggests that the interaction between the protein and anesthetic is not a traditional binding pocket with a complimentary positioning of residues but is more accurately described as an amphipathic “binding region” that has a relative energy minimum on a shallow energy landscape.

**Table 1:** **Desflurane contact probabilities** The desflurane contact probabilities were calculated for the bound-state simulation by recording is for each residue in the binding region there was any anesthetic atom within 3.5 Å of any protein atom, including backbone and side chains. This was done every 1 ns for the entire duration of the 100-ns simulation or until the anesthetic was judged to unbind (*i.e.*, more than 5 Å from protein). The data were collected for each subunit and then averaged over all five subunits to give the values presented here. The standard deviations presented are across all five subunits.

Residue	A	B	C	D	E	Mean contact probability (%)	S.D.
Tyr-119	10	23	90	71	14	41.6	36.4
Pro-120	28	22	97	98	33	55.6	38.4
Phe-121	61	10	25	44	90	46.0	31.2
Tyr-197	11	38	80	86	8	44.6	37.0
Ile-201	12	42	97	92	10	50.6	42.1
Ile-202	66	42	96	100	96	80.0	25.2
Met-205	16	35	77	81	28	47.4	29.7
Lys-206	58	14	3	4	87	33.2	37.6
Val-242	11	40	86	88	11	47.2	38.2
Tyr-254	66	8	30	57	94	51.0	33.1
Thr-255	23	45	97	97	43	61.0	34.0
Ile-258	67	36	70	88	98	71.8	23.8
Asn-307	51	3	2	4	67	25.4	31.2

Although the desflurane molecules are only loosely associated within the binding site suggested by the crystal structure, ligand unbinding and exit from the protein lumen is blocked by a group of bulky, mostly aromatic residues including Tyr-119, Phe-121, Tyr-197, Ile-201, Phe-238, Tyr-254, and Ile-259 (Fig. 2*d*). Recent experimental and computational studies (36, 41, 42) demonstrate that mutation of F238 to alanine (F238A or F14′A) switches the effect of anesthetics from inhibiting (the wild-type behavior) to potentiating GLIC, suggesting the bulky residues are critical for exerting the effects of anesthetics on ion channels. The steric bulk associated with the wild-type structure, however, is insufficient to fully stabilize the bound state; the interactions between the protein and anesthetic are loose enough that two desflurane molecules were observed to spontaneously dissociate from the protein during the 100-ns equilibrium simulation at 45 ns and 72 ns, respectively (Fig. 2, *a* and *b*). To exit the protein lumen, each anesthetic had to first diffuse vertically ∼8 Å toward the midplane of the lipid bilayer, where a small lateral opening exists between the Leu-206 (M1 helix) and Asn-307 (M4 helix) residues (Fig. 2*d*). The anesthetic then exits through this tunnel, which is firmly buried in the fatty acyl tail region of the membrane and, subsequently, after being fully released from the protein, repartitions into the glycerol region of the membrane where the minimum energy configuration has been previously described (43). Although it may be generally assumed that anesthetics unbind through water-accessible binding sites, here the exit of anesthetics from GLIC clearly proceeds through a membrane-embedded tunnel. The utilization of this tunnel helps explain why more effective anesthetics are generally quite hydrophobic, reflecting the initial findings of Meyer (1).

### Asymmetric binding of GLIC leads to a non-conductive channel

**Figure 3.**
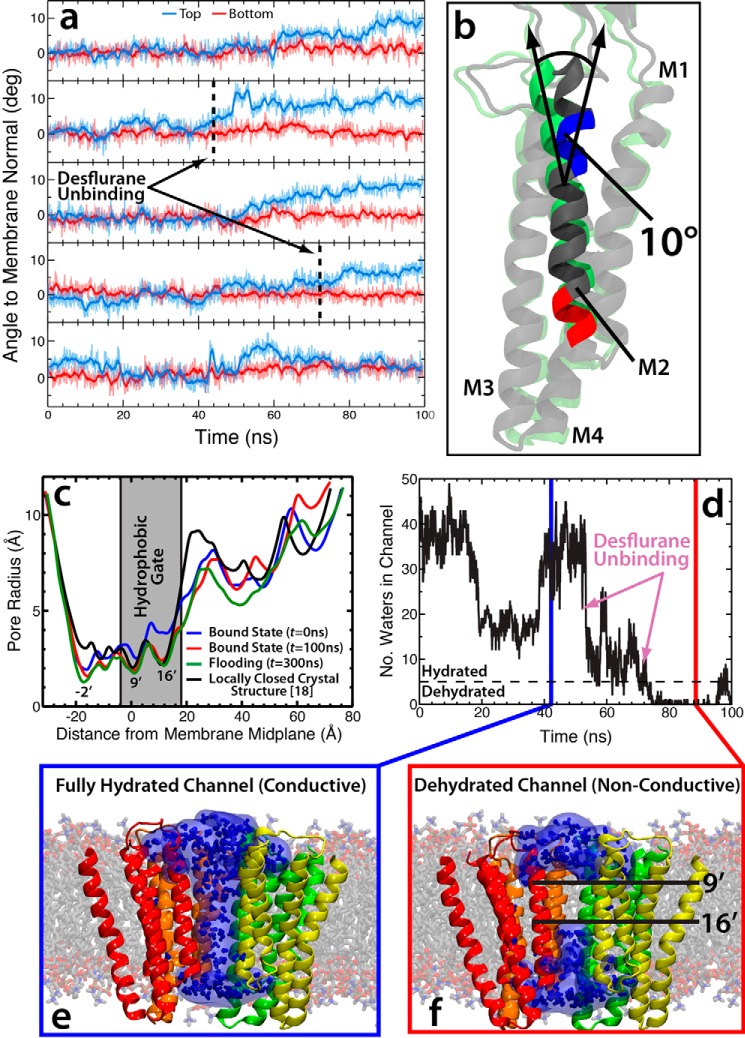
**Binding asymmetry of anesthetics induces conformational transition.** *a*, plots of the angles of the top (*blue*) and bottom (*red*) portions of the M2 helix for all five subunits in the simulation starting from the fully bound state. The *black dashed lines* in the *second* and *fourth plots* represent the time step where the desflurane exits the binding region in that subunit. *b*, molecular image showing the starting conformation (*green*) and asymmetrically bound conformation (*gray*) of the M2 helix. The *red* and *blue portions of the helix* shown in this image denote the residues used to measure the *top* and *bottom angles* in *a. c*, plots of the pore radius for the bound state simulation at *t* = 0 ns (*blue*) and *t* = 100 ns (*red*), the flooding simulation at *t* = 300 ns (*green*), and the locally closed crystal structure (*black*) (18) calculated using Hole (46). The −2′, 9′, and 16′ positions are labeled as well as the location of the presumed hydrophobic gate (*gray box*) where dehydration of the pore prevents ion conduction. *d*, number of water molecules in the hydrophobic gate (defined as Ile-233/Ile-240 or 9′/16′) as a function of time. The time points of desflurane unbinding in the bound state simulation is marked by the *arrows*, and the chosen hydration/dehydration cutoff (*n* = 5; Ref. 29) is shown as the *dashed black line. e* and *f*, molecular images showing the fully hydrated/conductive channel (*e*) and dehydrated/non-conductive channel (*f*). Only the TMD is shown, and one subunit has been removed for clarity. The protein is shown in schematic representation, whereas the water molecules are shown both as a surface, to demonstrate the gap in water, and as stick models.

Dissociation of desflurane from the crystal structure binding site leads to a detectable tilting of the upper portion of the M2 helices in a clockwise direction by ∼10°, causing an iris-like contraction of the pore radius and dehydration of the channel (Fig. 3) known to prevent ion conduction (24, 25). The unbinding of the two anesthetics yields an asymmetric binding pattern, which was hypothesized to be responsible for modulating channel function (44, 45) as previously observed from molecular simulations (37). These conformational changes, however, have not previously been directly linked with binding and unbinding of anesthetic from the crystal structure site. As can be seen in Fig. 3*c*, the asymmetrically liganded protein exhibits measurable contraction of the ion conduction pore radius by ∼1 Å (measured using Hole; Ref. 46) at the 9′ (Ile-233, *z* = −28) position and 2 Å at the 16′ (Ile-240, *z* = −18) position (see Fig. 1 for residue positions), which are thought to compose the boundaries of the hydrophobic gate for this channel (3, 25, 26, 29). The 9′ position is more constricted overall by 0.3 Å. Constriction of the pore at the hydrophobic gate (both 9′ and 16′) causes a dewetting of the channel, effectively closing the channel and preventing ion conduction (Fig. 3, *d* and *f*). Here, less than five water molecules in the hydrophobic gate region was considered dehydrated, based on previous structural studies showing this number of water molecules were needed for sodium ion translocation (24, 25, 29). There is not as large of a conformational change in the intracellular (lower) portion of the M2 helix (∼0.5 Å radius contraction of the −2′ position ring of glutamates compared with the equilibrated system) and little rearrangement of the extracellular domain, although it is very flexible and, thus, does not show a consistent profile across the simulations measured (Fig. 3).

The pore radius profile of the asymmetrically bound GLIC largely reproduces that measured for a crystal structure suggested to represent the “locally closed” state (18). One region where these profiles diverge is near the ring of glutamates that composes the selectivity filter at the intracellular mouth of the M2 helix (Fig. 3*c*). The wide opening at this region (*i.e.* −2′ position) is suspected to be an artifact of crystallization as even during equilibration of the symmetrically bound state in which anesthetics were harmonically restrained to their initial crystal structure positions, this region shows an immediate narrowing and constriction (Fig. 3*c*). Due to the uncertainty regarding the nature of the open and closed states of this and other ligand-gated ion channels (3, 26), the complete array of conformational changes induced by general anesthetics may not be fully appreciated, and it remains unclear whether the conformational changes observed here represent the canonical closed state of the channel or a distinct non-conductive state.

**Figure 4.**
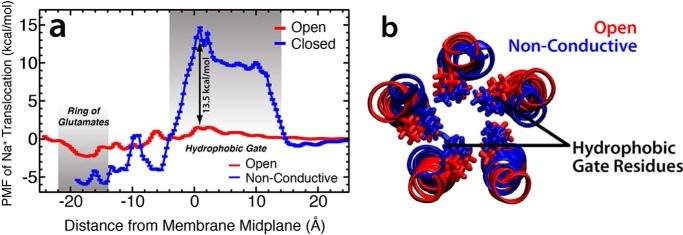
**Conformational transition of GLIC leads to non-conductance.** *a*, the PMF for translocation of a single Na^+^ ion across both the open (*red trace*) and non-conductive (*blue trace*) GLIC states. The energy was set to 0 for both curves at *z* = 25 Å where the Na^+^ ion was in bulk aqueous solution. Two areas are highlighted in *gray*: the hydrophobic gate demarcated by positions 9′ to 16′ (14 > *z* > −4 Å) and the ring of glutamates at position −2′ (−14 > *z* > −22 Å). *b*, the structures of the M2 helices of the open (*red*) and non-conductive (*blue*) states used in the umbrella sampling calculations. Shown in stick models are the isoleucine residues at positions 9′ and 16′ for the open and non-conductive states.

Conformational changes in GLIC structure that lead to dehydration and constriction of the central pore, as described above, should yield a channel that is no longer capable of conducting ions across the membrane (24, 25). To quantify the affect of anesthetic-induced conformational changes on ion conduction, umbrella sampling was used to calculate the free energy required to translocate a single Na^+^ ion across the TMD of GLIC in both the open state (17) and the structure of GLIC after the iris-like contraction representing the closed state (see “Experimental Procedures” for details on system configuration). The potential of mean force (PMF) profile (Fig. 4) illustrates that although the channel is open, there is only a small barrier to ion translocation of ∼1 kcal/mol at −27 < *z* < −29 Å. Moreover, there is a slight energy well at −45 < *z* < −51 Å of approximately −2 kcal/mol corresponding to the region containing the ring of glutamates that composes the selectivity filter for cation-permeable channels and aid in conduction (27–29). After the conformational changes associated with asymmetric binding of anesthetics, however, a significant energy barrier of ∼15 kcal/mol arises in the hydrophobic gate of the GLIC channel (Fig. 4*a*). This equates to a barrier ∼13.5 kcal/mol greater than for the open state, making the channel functionally non-conductive.

### A novel transmembrane binding site identified from flooding simulations

**Figure 5.**
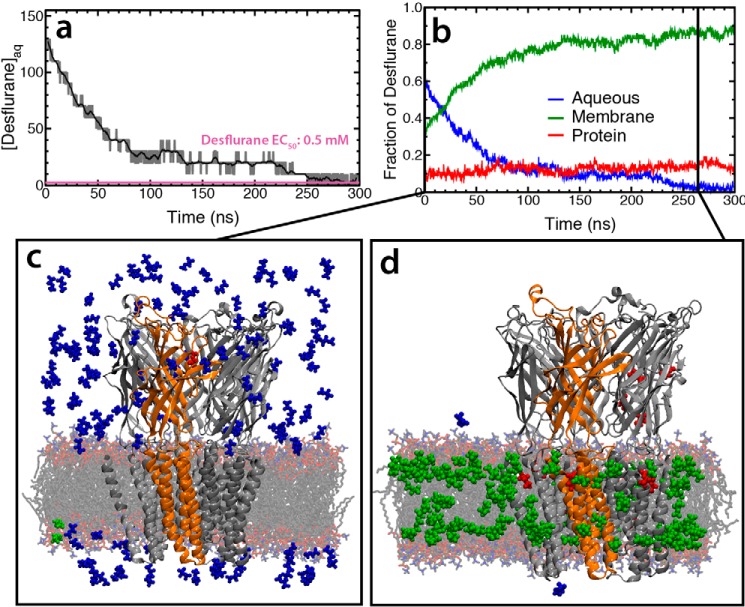
**Partitioning of desflurane into the membrane and GLIC in flooding simulation.** *a*, aqueous concentration of desflurane as a function of simulation time. By the end of the simulation there was a fluctuation between 0 and 2 molecules of desflurane in aqueous solution that correlates to a fluctuation between 0 mm and 10 mm. However, the concentration could not have a value between 0 and 10 mm due to the size limits of the system. *b*, fraction of desflurane molecules in each phase (*blue*, aqueous; *green*, membrane; *red*, protein). It is clear that most of the molecules partition to the membrane within the first 100 ns. Snapshots of the system at the beginning (*c*) and end (*d*) of the simulation show the positions of every desflurane molecule in the system. The color of the desflurane molecule corresponds to its environment (*blue*, aqueous; *green*, membrane; *red*, protein).

**Figure 6.**
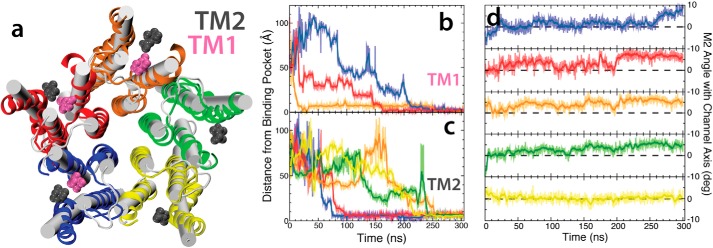
**Identification of a distinct binding region from flooding simulations.** *a*, snapshot of the TMD of GLIC showing the initial (*white cylinders*) and final states (*multicolor schematic*) of the flooding simulation together with the TM1 (*magenta*) and TM2 (*gray*) binding regions. Only three of the five TM1 sites were occupied during the simulation, whereas all five TM2 sites were occupied. *b* and *c*, plots of the distance between the bound desflurane and the TM1 site (*b*) and TM2 site (*c*) are shown with the running average (averaged >30 ns) as the thick curve and the raw position data as the light trace. The colors in these plots correspond with the color of the subunits shown in *a. d*, plots of the M2 angle with the channel axis for each of the five subunits. The colors in these plots correspond with the color of the subunits shown in *a*.

The simulations described above focused on the anesthetic binding region identified from published X-ray crystal structures (17); however, it is possible that other functionally relevant binding sites are present or accessible from these structures. The existence of such sites was queried using five independent 300-ns “flooding” simulations in which 150 copies of the anesthetic were added to the solution phase of a simulation system containing unbound GLIC (see Ref. 32 for more details). Almost immediately, the anesthetic molecules began to partition into the membrane (Fig. 5), ultimately leaving between zero and two anesthetics in aqueous solution by the end of each simulation. To ensure the structural stability of the simulation systems, the membrane width and backbone RMSD of the protein was calculated (supplemental Fig. S1). The RMSD of the protein remained <3 Å for the entire duration of all simulations, with the average RMSD for each simulation being <2.5 Å. Moreover, the membrane width for all simulations converged to an average of 37.31 Å. Based on these criteria, the simulations were deemed to be stable. Multiple binding areas, defined by protein residues that interact with anesthetic for >50 ns of the simulation, were identified. Of these potential binding sites, only two were observed across more than one subunit in multiple simulations (Fig. 6), namely the same region as identified in the crystal structure (17, 47) (called TM1 herein) and a second transmembrane binding site (TM2) located outside of this region, which has not been previously described. All other protein residues that establish long-lived interactions with anesthetic molecules most likely represent nonspecific adhesion of the anesthetic to the protein surface rather than a functional binding site.

As opposed to the previous set of simulations, in which desflurane spontaneously dissociates from the TM1 site, here we observe spontaneous association of desflurane to the TM1 site across multiple simulations (supplemental Fig. S2). In each of these simulations, the path to the TM1 site is the same as that presented above. Namely, desflurane molecules in aqueous solution first partition to the glycerol region of the membrane. Once partitioned to the membrane, the anesthetic must move close to the membrane center (supplemental Fig. S2) to access the membrane-embedded entrance (Fig. 2*d*), which lies ∼3 Å from the membrane center. Here, the anesthetic encounters residues Leu-206 and Asn-307, which afford an opening to the protein lumen. After this, desflurane is free to move within the intrasubunit lumen to interact with the TM1 site as detailed above (Fig. 2*d*). Therefore, from these simulations together with those presented above, the association and dissociation of anesthetics from the TM1 site proceeds through the same pathway.

Occupancy of the newly identified TM2 site was observed in all five subunits across the complete set of simulations. Building upon previous studies characterizing anesthetic partitioning into the membrane (43), it was notable to that anesthetic entry and binding to both sites occurred exclusively by diffusion from within the membrane. Indeed, similar to accessing the TM1 site, anesthetics that interact with the TM2 site must first partition to the glycerol region of the membrane (43) and then diffuse further toward the membrane center. Once close to the membrane center (supplemental Fig. S3), desflurane can interact with the Asn-307 residue that contributes to the TM2-binding pocket. In fact, many molecules that bind to the TM1 site interact with the TM2 site first, as the TM2 site is along the path from membrane to TM1 binding site. Furthermore, the occupation of the TM2 site appears to affect the dynamics of the anesthetic bound to the TM1 site. As can be seen in Fig. 6, one subunit (red) has a desflurane molecule stably bound to TM1 for >150 ns while the TM2 site is occupied. In contrast, when no desflurane is bound to the TM2 site, as in the case of the orange subunit, binding to the TM1 site is unstable. In this latter case, a desflurane molecule occupies the TM1 site for only 12 ns before dissociating from GLIC completely.

**Figure 7.**
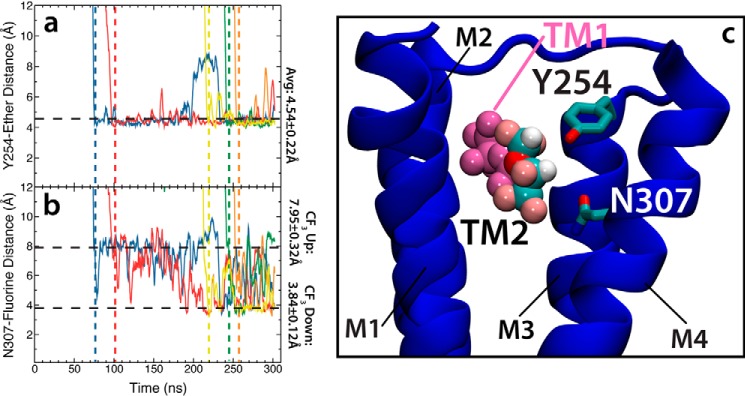
**Anesthetic-protein interactions at the newly identified TM2 site.** *a*, plot of the distance between the hydroxyl group of Tyr-254 and the ether of the desflurane molecule bound to the TM2 site. Each color represents a different subunit, and the *vertical dashed lines* represent the time point at which the desflurane binds to the TM2 site in each subunit. The color of the dashed lines correlates with the color of the distance trace. *b*, plot of the distance between the trifluoromethyl (CF_3_) group of the desflurane bound to the TM2 site and the NH_2_ moiety of the Asn-307 amide. The *vertical dashed lines* represent initial binding of desflurane to the TM2 site. *c*, molecular image of the TM2 site with desflurane bound (multicolor van der Waals) and a desflurane molecule occupying the TM1-binding region (*magenta* van der Waals). Here, the protein backbone is shown in blue and Y254/Asn-307 are shown as multicolor stick models. Other subunits and the membrane are omitted for clarity.

The effect communicated from TM2 to TM1 appears to be due to an interaction between the anesthetic in the TM2 site with Asn-307 (identified as a critical residue lining the exit tunnel in this study), partially blocking exit from the TM1 site. This suggests that by acting as a steric barrier to dissociation, occupancy of the TM2 site might increase occupation of the TM1 site, thereby magnifying the effect of the anesthetic on the ion channel. Anesthetic binding to the TM2 site appears to be more long-lived than observed for the TM1 site, as some subunits are occupied for >200 ns. The desflurane molecule in TM2 is held in place by a hydrogen bond between Tyr-254 and the ether oxygen of desflurane as well as by an interaction between the electronegative fluorine atoms and Asn-307 (Fig. 7). Although the interaction between Tyr-254 and the anesthetic is stable, the interaction between Asn-307 and the anesthetic is weaker. In fact, the anesthetic was observed to rotate within this site such that each end of the desflurane molecule interacts multiple times with the amide of Asn-307 throughout the simulation (Fig. 7*c*). Calculation of the root mean squared fluctuation for each desflurane bound to a TM2 site (Table 2) produces an average of 4.11 ± 1.35 Å, demonstrating the labile nature of this interaction. The presence of multiple bound anesthetics per subunit, in addition to those that appear to bind non-specifically to the protein, helps explain why such high concentrations of anesthetic are required to induce clinical effects when compared with other drugs. Anesthetics, in fact, seem to be indiscriminate, and it has become more clear that these drugs bind to multiple targets across the nervous system (48).

**Table 2:** **Fluctuation of desflurane in the TM2-binding pocket** For each replicate simulation system, the number of times a desflurane molecule bound to the TM2 site along with the average (±S.D.) amount of time each desflurane molecule occupied the TM2 site was recorded and is presented here. Although bound to the TM2 site, the distance between the desflurane ether oxygen and the Tyr-254 hydroxyl oxygen was measured every 0.2 ns, and the average distance (±S.D.) was calculated across all binding events in a simulation. The average root mean square fluctuation (RMSF) of each bound desflurane within the TM2 binding site was calculated using the average structure of a bound desflurane as the reference. The RMSF of each desflurane deemed bound to the TM2 site was recorded every 0.2 ns for the duration of binding and time-averaged. For each replicate, the minimum and maximum RMSF for all bound desflurane molecules is presented along with an average (±S.D.) RMSF for all bound desflurane in that replicate. Using data from all replicates, an average (±S.D.) RMSF, Tyr-desflurane distance, and time spent in the TM2 site was calculated.

Replicate	No. binding events	Time in pocket	Tyr-desflurane distance	Minimum RMSF	Maximum RMSF	Average RMSF
		*ns*	Å	Å	Å	Å
1	5	127.5 ± 84.6	4.54 ± 0.22	2.78	4.90	3.73 ± 0.79
2	6	88.4 ± 79.5	4.31 ± 0.38	3.35	6.01	4.41 ± 1.10
3	5	34.4 ± 41.7	4.10 ± 0.15	2.67	3.92	3.30 ± 0.48
4	8	96.9 ± 70.3	4.25 ± 0.29	2.70	3.76	3.52 ± 0.38
5	8	53.4 ± 48.0	4.67 ± 0.80	3.09	8.34	5.28 ± 1.99
All	32	79.2 ± 68.7	4.36 ± 0.52			4.11 ± 1.35

**Figure 8.**
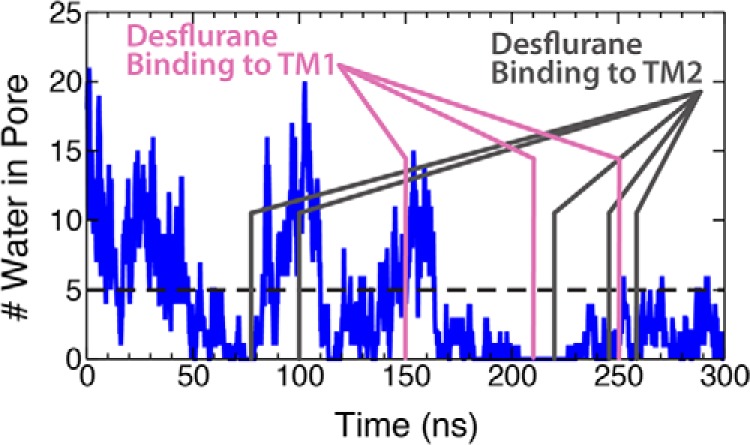
**Dehydration of the GLIC ion pore in flooding simulation.** Plot of the number of water molecules in the hydrophobic gate in the flooding simulation. Here, the *three magenta lines* represent the time points when desflurane binds to the TM1 sites discussed previously. Here, dehydration is considered to be <5 water molecules in the hydrophobic gate as this is the number required to transport a Na^+^ (29).

Consistent with the simulations starting from the fully bound state described above, asymmetric binding observed during the flooding simulations causes a tilting of the upper portion of the M2 helices (Fig. 6) leading to an iris-like contraction and dehydration of the pore (Figs. 3 and 8). Quantitative comparison of the contracted and dehydrated states, arriving from either a fully bound or unbound starting state, by measuring the RMSD between the protein structures taken from the final frame of the simulations, yields an average RMSD of 0.99 ± 0.08 Å. Moreover, the profiles for the pore radius from these simulations are nearly identical within the TMD. Due to this strong similarity in structure, it is reasonable to believe that the structure induced by anesthetic binding in the flooding simulations (Fig. 6*d*) would also be non-conductive. Indeed, recent crystal structures of GLIC mutants that have shown to be non-conductive via electrophysiological studies (49) bear striking structural similarity to the non-conductive state obtained through the simulations described in this study (Table 3), with an RMSD of <1.5 Å in the TMD and <1.3 Å in the pore-lining helices.

**Table 3:** **Structural similarity of simulated and crystallographic non-conductive states** Measured RMSD between the crystal structure of a non-conductive state of GLIC (49) and the non-conductive states observed in the bound state and flooding simulations. RMSD for the bound state simulation is measured every 0.1 ns over the last 20 ns of the trajectory, whereas the RMSD for the flooding simulation is measured every 0.1 ns over the last 50 ns of the trajectory. In both cases the crystal structure (PDB 4LMK) was used as the reference structure, and the appropriate backbone atoms were matched to those of the crystal structure. The data presented here are in the form of mean ±S.D. ECD, extracellular domain.

Structure	Bound state RMSD	Flooding RMSD
	Å	Å
Protein	2.97 ± 0.05	2.87 ± 0.22
ECD (14–192)	3.08 ± 0.16	3.21 ± 0.29
TMD (195–315)	1.49 ± 0.06	1.42 ± 0.05
Pore (221–244)	1.20 ± 0.04	1.30 ± 0.05

**Figure 9.**
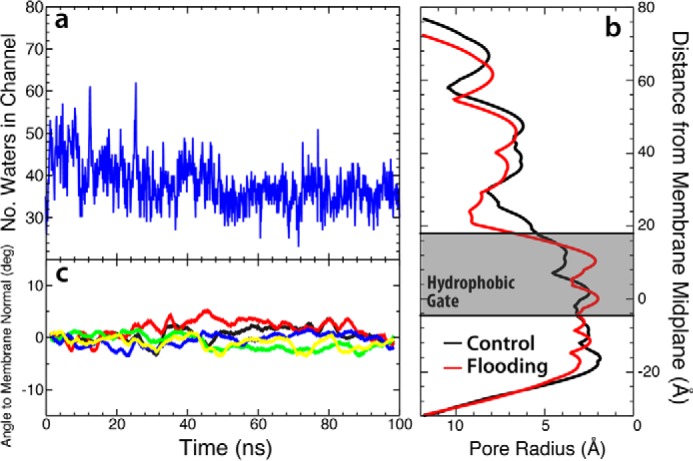
**Structure of GLIC channel in control simulation.** *a*, plot of the water number in the hydrophobic gate as a function of simulation time. The number of waters in the hydrophobic gate remains constant throughout the simulation. *b*, plot of pore radius for both the control/anesthetic-free simulation (*black*) and the flooding simulation (*red*) found using the HOLE program (46). Presented here is the final frame of each simulation. *c*, plot of the angle between the M2 helix and the membrane normal as a function of time for each subunit, similar to Fig. 3*a*. The colors in this plot correspond to those of the individual subunits seen in Fig. 2*a*. Each trace is presented as a running average of the data in windows of 5 ns.

There is some concern regarding the stability of the crystal structure and whether the open state remains hydrated (31, 35). Performing a control simulation of GLIC without desflurane shows that the hydrophobic gate remains hydrated for >100 ns (Fig. 9). Moreover, the ion pore radius of the control simulation is not as contracted as that of the flooding simulation and resembles more that of the open configuration of the crystal structure (Fig. 9), whereas the conformational changes observed in the flooding simulations as well as the initially bound simulation are not observed in the control. Therefore, the dehydration due to conformational changes observed in our simulations is likely not from a defect in the crystal structure, but rather, is an effect from anesthetics interacting with the ion channel.

### Y254A mutant demonstrates decreased anesthetic binding

**Figure 10.**
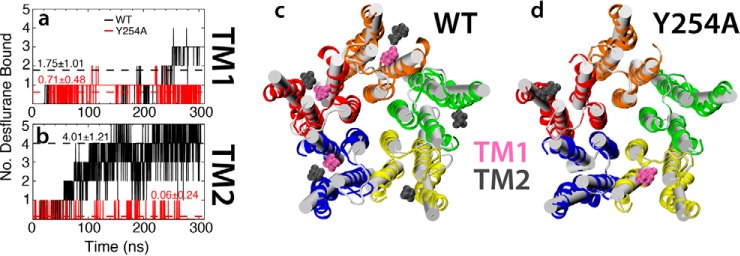
**Y254A mutant decreases anesthetic interaction with the TM1-binding region.** Plots of the number of desflurane molecules bound to the TM1 (*a*) and TM2 (*b*) sites over the duration of the simulation are shown for both the wild-type (*black trace*) and Y254A mutant (*red trace*). The *dashed lines* represent the average of the entire simulation, with the numbers representing the mean ± S.D. Molecular images of the TMD of both the wild type (*c*) and Y254A mutant (*d*) are shown, with the initial state displayed in *white schematic* and the final state displayed in *multicolor ribbons*, each subunit shown in a different color. In both images a representative snapshot of the system is shown with all anesthetics bound to the TM1 (pink van der Waals surface) and TM2 (*gray* van der Waals surface) regions.

Observing the effects of high concentrations on anesthetic binding to GLIC revealed a novel transmembrane binding site, termed TM2, that appears to affect the affinity of desflurane for the crystallographically identified TM1 site. Desflurane binding to the TM2 site is mostly afforded through an interaction between the hydroxyl group of Tyr-254 and the ether oxygen of the anesthetic (Fig. 7). To test the importance of this interaction on binding to the TM2 site and how it may affect binding within the TM1 site, a 250-ns flooding simulation was performed for the Y254A mutant (Fig. 10). This mutation clearly abolishes binding to the TM2 site as the average number of bound desflurane molecules decreased from 4.01 ± 1.21 to 0.06 ± 0.24 in wild type and Y254A mutant simulations, respectively. Thus, it appears that Tyr-254 represents a critical interaction necessary to stabilize desflurane binding to the TM2 site. Abolishing anesthetic binding to the TM2 site was also observed to impair binding to the TM1 site, decreasing the average number of bound molecules from 1.75 ± 1.01 to 0.71 ± 0.48 in wild type and Y254A mutant simulations, respectively, suggesting that binding to TM1 and TM2 might be correlated, although a more quantitative establishment of this correlation would require a significantly larger data set.

### Conclusions

Despite extensive studies, the questions of where anesthetics bind to ion-permeable channels and how they exert their effects on the dynamics of these channels remains unclear. Molecular dynamics simulations offer unparalleled resolution and atomic detail that can probe how anesthetics interact with ion channels. Using newly developed parameters for desflurane (43), simulations totaling 2.75 μs were performed to gain a deeper understanding of anesthetic binding and effect on GLIC, a prokaryotic homologue of mammalian Cys-loop receptors. From simulations starting from the fully bound state, it was observed that desflurane is only loosely bound to the TM1 site, an intrasubunit site described in the crystal structure (17), even allowing spontaneous dissociation from the protein in <100 ns of simulation. Moreover, from so-called flooding simulations, a second binding site was revealed, termed the TM2 site, that appears to affect the dynamics of desflurane anesthetics bound to the TM1 site. In fact, a point mutation, Y254A, that abolishes binding of desflurane to the TM2 site significantly decreased the amount of anesthetic bound to the TM1 site (Fig. 10) and, possibly, the anesthetic potency of desflurane. Thus, it appears that double occupation of the intrasubunit lumen by anesthetic is important for anesthetic action. Although beyond the scope of this study, experiments looking at the effect of the Y254A mutant on anesthetic affinity for the GLIC channel should be performed to corroborate the findings discussed herein. The loose binding of desflurane together with the need for multiple anesthetics bound per subunit and the large number of anesthetics absorbed by the membrane provide insight into why such a large concentration of anesthetic is needed to observe a clinical effect.

In addition to the double-occupancy model, the simulations described herein were able to establish that anesthetic binding/unbinding proceeds through a membrane-embedded pathway. Although flooding simulations showed that anesthetics preferentially partition to the membrane before binding to GLIC (Fig. 5), the simulations of fully bound states showed that the anesthetics diffuse into and out of the protein via an opening afforded by Leu-206 and Asn-307, located at the interface between the membrane and the intrasubunit lumen. The need for membrane association prior to membrane binding demonstrates why more hydrophobic molecules make better anesthetics.

Lastly, upon induction of an asymmetric binding pattern, a subtle structural change in which the M2 helices of GLIC undergo a tilt into the central pore by 10°, results in an iris-like contraction and subsequent dehydration of the channel near the hydrophobic gate (Fig. 3). Although it is currently unclear what causes this conformational change, the same change is observed in both simulations starting from fully bound or unbound states, and the resulting structures are very similar to a recently published, inactivated channel (Table 3) (49). Using the initial open structure (17) and the structure garnered after constriction, umbrella-sampling calculations were employed to better understand how conformational changes affected the landscape of ion translocation. Whereas a relatively flat free energy profile is observed for the open state, meaning ions should flow freely across the channel when activated, the conformational changes, including the constricted state, led to an increase in the barrier to ion translocation by 13.5 kcal/mol, making Na^+^ ion conduction improbable. Although this is not a definitive closed state of this channel, the fact that ion translocation would be impossible in this state together with the similarity of the structures obtained here with those of inactive GLIC channels strongly suggests that this state may be a reasonable representation of the closed state.

## Experimental procedures

### Preparation and membrane-embedding of the anesthetic-bound GLIC system

Using the X-ray crystal structure of GLIC bound with general anesthetic (PDB code 3P4W) (17), the ion channel together with the five bound desflurane molecules was placed in a POPC bilayer measuring 115 × 115 Å^2^ in the *xy* plane using the Membrane Builder plugin of VMD (50). Although this protein is naturally embedded in bacterial membranes predominantly composed of POPE (1-palmitoyl-2-oleyl-*sn*-glycero-3phosphoethanolamine) and POPG (1-palmitoyl-2-oleyl-*sn*-glycero-3-phosphoglycerol) lipids, previous computational studies embedded GLIC into a POPC membrane (31, 32, 42) more typical of mammalian cells, a design feature that is followed here. After GLIC was embedded in the membrane, the entire system was solvated with 150 mm NaCl (aqueous) solution, yielding a molecular system of 180,000 atoms and initial dimensions of ∼115 × 115 × 132 Å^3^. The coordinates for the system were then uploaded to the PROPKA 3.1 plugin for VMD (51) and the p*K_a_* shifts calculated for each amino acid accounting for the local environment (*i.e.* membrane, water, and ions). If the estimated p*K_a_* of a side chain was >4.0, the pH_50_ of GLIC (12, 23), then the residue was assumed to be protonated in the open state during the simulation. This cutoff was chosen due to the fact that the protein was crystallized at pH 4.0 and was interpreted to be in the open configuration (17). The residues protonated were Glu-26, Glu-35, Glu-67, Glu-75, Glu-82, Asp-86, Asp-88, and Glu-177, in accordance with previous simulation studies (15, 32).

Once the system was constructed, the lipid bilayer was relaxed in two steps while harmonically restraining all heavy atoms of the protein and anesthetic molecules (*k* = 5.0 kcal/mol/Å^2^) yet allowing water molecules and ions to diffuse freely. The lipid tails were first “melted” for 2 ns while the head group atoms (*i.e.* choline, phosphate, and glycerol backbone) were harmonically restrained (*k* = 5.0 kcal/mol/Å^2^). After this, the restraints on the head group atoms were released, and the whole bilayer was relaxed for 2 ns. Upon full relaxation of the POPC bilayer, the restraints on residue side chains and anesthetic ligands were removed, and the system was further simulated for 5 ns to equilibrate the anesthetic binding region. Finally, all remaining restraints on the protein were released, and the entire system was equilibrated for 5 ns, which resulted in final dimensions of ∼116 × 115 × 130 Å^3^ for the full system. The resulting structure was simulated for 100 ns.

### Construction of the apoGLIC system and flooding simulations

To probe for additional binding sites beyond those observed in the GLIC crystal structure (17), the flooding technique (32, 52–54), simulations in which a large number of copies of the drug (desflurane) are placed in aqueous solution and allowed to diffuse freely throughout the system to find areas of low energy, were employed for the apo form of the GLIC channel. The advantage of this method is that the high copy number allows extensive exploration of configurational space on time scales that are currently achievable through conventional MD simulations. Using the equilibrated membrane-bound system described above, the five desflurane molecules were removed from the system, and the system was equilibrated for 5 ns while restraining the protein backbone (*k* = 5.0 kcal/mol/Å^2^) to allow for the side chains near the anesthetic binding region to relax after removal of desflurane. The harmonic restraints were subsequently removed, and the entire system was allowed to further relax during an additional 5-ns simulation. The resulting system with dimensions of ∼116 × 115 × 130 Å^3^ was used in all flooding simulations.

Anesthetic was added to the equilibrated apo channel by randomly placing 150 desflurane molecules in the aqueous solution at a ratio of 1 desflurane per 2 POPC lipids while ensuring that no anesthetic molecule was within 10 Å of any protein atom or 5 Å of any lipid atom. This ratio gave an initial aqueous desflurane concentration of 150 mm, much larger than the EC_50_ of 0.56 mm (13); we note, however, the goal of using such a high concentration is to increase the probability of finding an anesthetic binding site during the simulations. Once all of the anesthetic molecules were placed, any water molecules within 2.5 Å of a desflurane molecule were removed from the system to prevent steric clashes. A short equilibration simulation of 2 ns was performed in which the desflurane molecules, the lipids, and all protein atoms were harmonically restrained (*k* = 5.0 kcal/mol/Å^2^). Five total production simulations were then performed on the fully unrestrained system, each lasting >300 ns.

### Introducing a Y254A mutation into the GLIC system

To test the role of double occupancy in stabilizing bound anesthetics, a Y254A mutation was introduced with the expectation of abolishing anesthetic binding to the TM2 site. Starting from the equilibrated apo system described above, the Tyr-254 residue was mutated to alanine in all five subunits using the Psfgen plugin of VMD (50). All heavy atoms of the protein, except the newly mutated residues, were harmonically restrained (*k* = 5.0 kcal/mol/Å^2^), whereas the mutated residues were allowed to explore conformational space over a 5.0-ns simulation. After this, a 5.0-ns simulation without restraints was performed to allow changes in the remainder of the binding region to adjust to the newly mutated residues. The equilibrated Y254A system was then used for production flooding simulations (300 ns) performed using the same protocol as for the wild type apo system.

### Computing free energy of ion translocation

To more quantitatively characterize the ion conduction states of the channel, umbrella sampling was used to calculate PMF profiles associated with the translocation of a Na^+^ ion across the GLIC channel in the open- and in the non-conductive states. The non-conductive state was taken to be the final frame of the 100-ns simulation where desflurane was initially bound to GLIC per the crystal structure (17). A total of 50 umbrellas was placed at 1 Å intervals between the biasing potential centers, extending along the membrane normal from *z* = −5 (∼5 Å above the TMD) to *z* = −55 (∼10 Å below the ring of glutamates at −2′). The center of mass for a Na^+^ ion was restrained to the *z* coordinate of each umbrella center by a harmonic potential of *k* = 7.17 kcal/mol/Å^2^. Each umbrella was minimized for 5000 steps and simulated for 10 ns, recording the position of the molecule every 0.2 ps (a total of 50,000 frames per umbrella). Throughout the calculations, the backbone atoms of the pore-lining M2 helices were restrained (*k* = 5.0 kcal/mol/Å^2^) to their initial positions to ensure the channel remained in either an open or a non-conductive state. The PMF was reconstructed using the weighted analysis histogram method (WHAM) (55, 56) from the last 9.5 ns of the trajectories. This protocol was performed for both the open channel (17) and the non-conductive state obtained from the simulations reported here.

### General simulation protocols

All simulations were performed using NAMD2 (57) with the set of force-field parameters previously developed for desflurane (43) and the CHARMM36 (58, 59) parameters for protein and POPC lipids. The desflurane parameters used here have been tested exhaustively and shown to reproduce important physical properties, including enthalpy of vaporization and free energy of solvation in multiple environments (43). The TIP3P model (60) was used for explicit water molecules. The target pressure was set to 1.0 atm, the temperature of the system was set to 298 K (25 °C), and the time step was 2.0 fs. Constant pressure was maintained using the Noœe-Hoover Langevin piston method (61, 62). A Langevin damping coefficient (γ) of 1 ps^−1^ was used to maintain the temperature of the system. Non-bonded interactions were cut off after 12 Å with a smoothing function applied after 10 Å. Long-range electrostatic interactions were treated using the particle mesh Ewald method (63) with a grid density >1 Å^−3^. Bonded and non-bonded forces were calculated at every time step, whereas particle mesh Ewald calculations were performed at every other time step.

### Structural analysis of simulated GLIC channels

Asymmetry in the binding of anesthetics to GLIC is reported to cause structural and conformational changes (37). In the present study several metrics have been used to quantitatively assess and characterize these changes and will be briefly discussed here to promote overall clarity of the paper. To map the path desflurane traverses to enter and exit the protein, contact probabilities were computed between desflurane and each residue in the TMD. Desflurane was defined to be in contact with a residue if it was within 3.5 Å of any heavy atom of the residue. A contact event was registered for each frame in which desflurane was observed within the contact distance of a specific residue. The contact probability was computed for each residue by dividing the total number of contact events by the total number of frames. In response to an asymmetric ligand occupancy, structural changes, including tilting of the M2 helices, ion pore constriction, and dewetting of the channel, were observed in the present study, replicating the observations of a previous study (37). To measure the tilt of the M2 helices, four-residue turns were chosen at the intracellular, middle, and extracellular portion of each M2 helix. For each frame of the simulation, a vector was drawn between the center of mass of the intracellular and middle residues as well as between the middle and extracellular residues. The angle between each helix vector and the membrane normal was then calculated and plotted over the simulation time. Tilting of the M2 helices toward the center of the channel leads to constriction of the ion pore; this constriction was quantitated using Hole (46) to measure the pore radius. Lastly, to measure how hydration of the ion pore was affected by these structural changes, the number of water molecules within the hydrophobic gate (between the 9′ and 16′ residues) was calculated. As the initial radius of the hydrophobic gate after equilibration was found to be 4 Å, a cylinder defined to have a radius of 4 Å and a height measuring the distance measured between the Cβ atoms of Ile-233 (9′ residue) and Ile-240 (16′ residue) was defined for each frame. The number of water molecules contained within this cylinder was then plotted as a function of time to determine the time-dependent hydration of the channel.

## Author contributions

M. J. A. and E. T. conceived and designed the study. M. J. A. and C. G. M. performed the data acquisition. M. J. A. and C. G. M. performed data analysis and interpretation. M. J. A., C. G. M., and E. T. prepared the manuscript for submission. All authors reviewed the results and approved the final version of the manuscript.

## Supplementary Material

Supplemental Data

## Footnotes

2 The abbreviations used are:

pLGIC: pentameric ligand-gated ion channel
GLIC: *G. violaceus* ligand-gated ion channel
PMF: potential of mean force
POPC: 1-palmitoyl-2-oleyl-*sn*-glycero-3-phosphocholine
RMSD: root mean squared distance
RMSF: root mean squared fluctuation
TM1: transmembrane binding site 1
TM2: transmembrane binding site 2
TMD: transmembrane domain.
