# Supplementary material for: A membrane-embedded pathway delivers general anesthetics to two interacting binding sites in the Gloeobacter violaceus ion channel
Source: J Biol Chem. 2017 Apr 18;292(23):9480–92. doi: 10.1074/jbc.M117.780197 (PMC5465477; doi:10.1074/jbc.M117.780197)
Supplement: Supplemental Data [file supp_292_23_9480__index.html]

A membrane-embedded pathway delivers general anesthetics to two interacting binding sites in the Gloeobacter violaceus Ion Channel — A membrane-embedded pathway delivers general anesthetics to two interacting binding sites in the Gloeobacter violaceus ion channel — Membrane-mediated general anesthetic binding to GLIC — Supplemental Data 

# A membrane-embedded pathway delivers general anesthetics to two interacting binding sites in the *Gloeobacter violaceus* ion channel

## Supplemental Data

- SI (.docx, 2.8 MB) - Word version of the revision 2 SI material
- SI (.pdf, 1.8 MB) - PDF version of the revision 2 SI material
