# Supplementary material for: A membrane-embedded pathway delivers general anesthetics to two interacting binding sites in the Gloeobacter violaceus ion channel
Source: J Biol Chem. 2017 Apr 18;292(23):9480–92. doi: 10.1074/jbc.M117.780197 (PMC5465477; doi:10.1074/jbc.M117.780197)
Supplement: Supplemental Data [file 10.1074_M117.780197_jbc.M117.780197-1.docx]

# A Membrane–Embedded Pathway Delivers General Anesthetics to Two Interacting Sites in the *Gloeobacter violaceus* Ion Channel

Mark J. Arcario, Christopher G. Mayne, and Emad Tajkhorshid

Table of Contents:

Fig. S1 – Structural Stability of the Simulation Systems . . . . . . . . . . . . . . . . . . . . S2

Fig. S2 – Binding Pathway of Desflurane to TM1 Site . . . . . . . . . . . . . . . . . . S3–S4

Fig. S3 – Binding Pathway of Desflurane to TM2 Site . . . . . . . . . . . . . . . . . . S5–S6


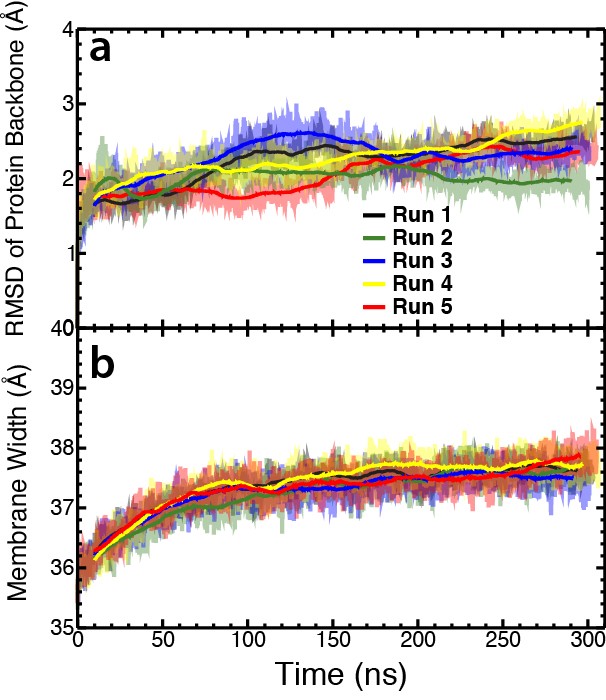


**Figure S1: Structural Stability of the Simulation Systems.** (a) Plot of the RMSD of the backbone atoms of the entire protein as a function of time is presented for all replicates (labeled Runs 1-5 in the plot) with the initial configuration used as the reference structure. The transparent curves in this plot represent raw data sampled every 0.2 ns. The solid curves represent the same data with a running average of 20 ns. (b) Plot of the membrane width as a function of time is presented for all replicates (labeled Runs 1-5). The width was measured every 0.2 ns and was taken to be the distance between the center of mass of the phosphate layer of the *cis*and *trans*leaflets. The transparent curves represent raw data, while the solid curves represent a running average of 20 ns.


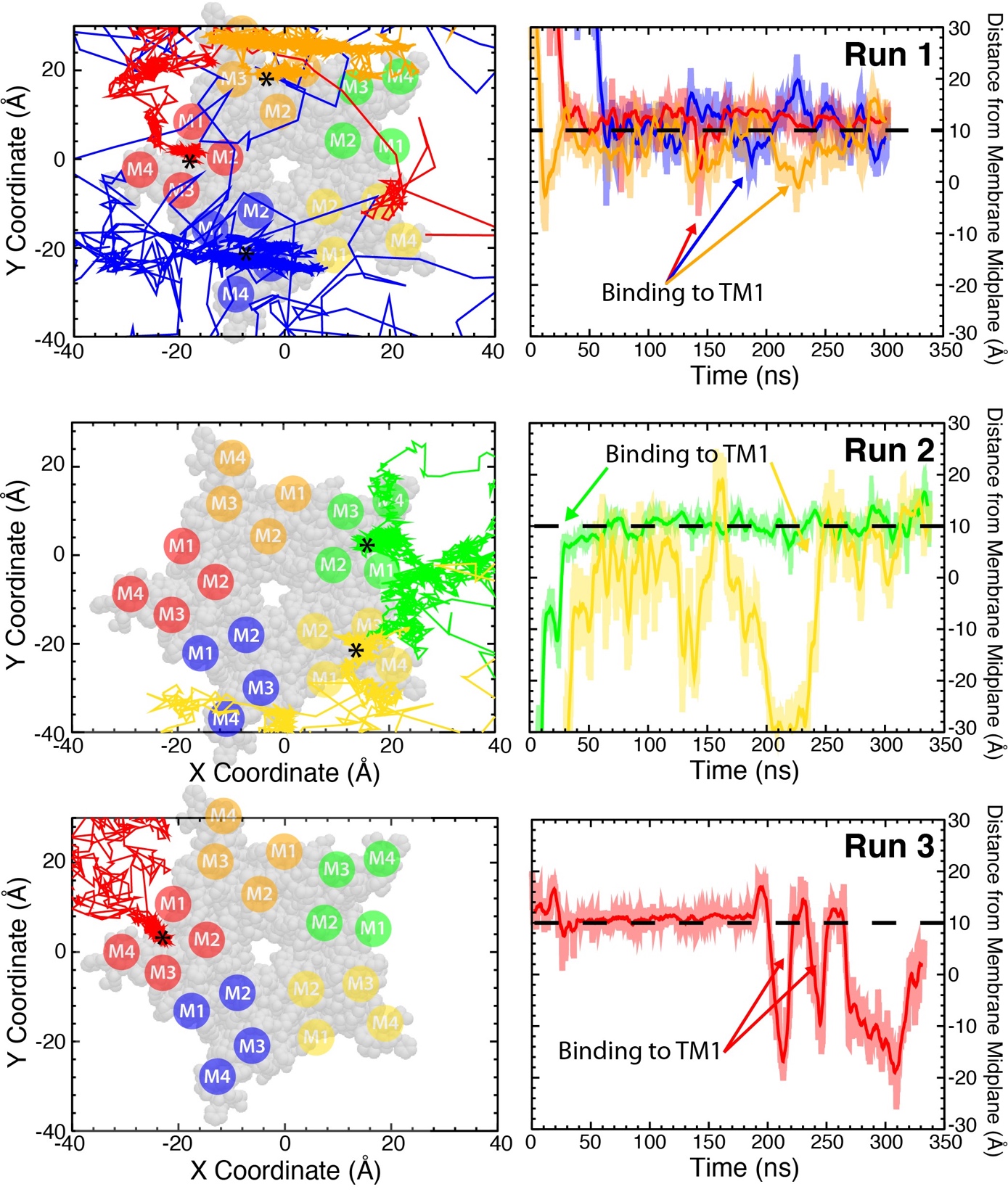


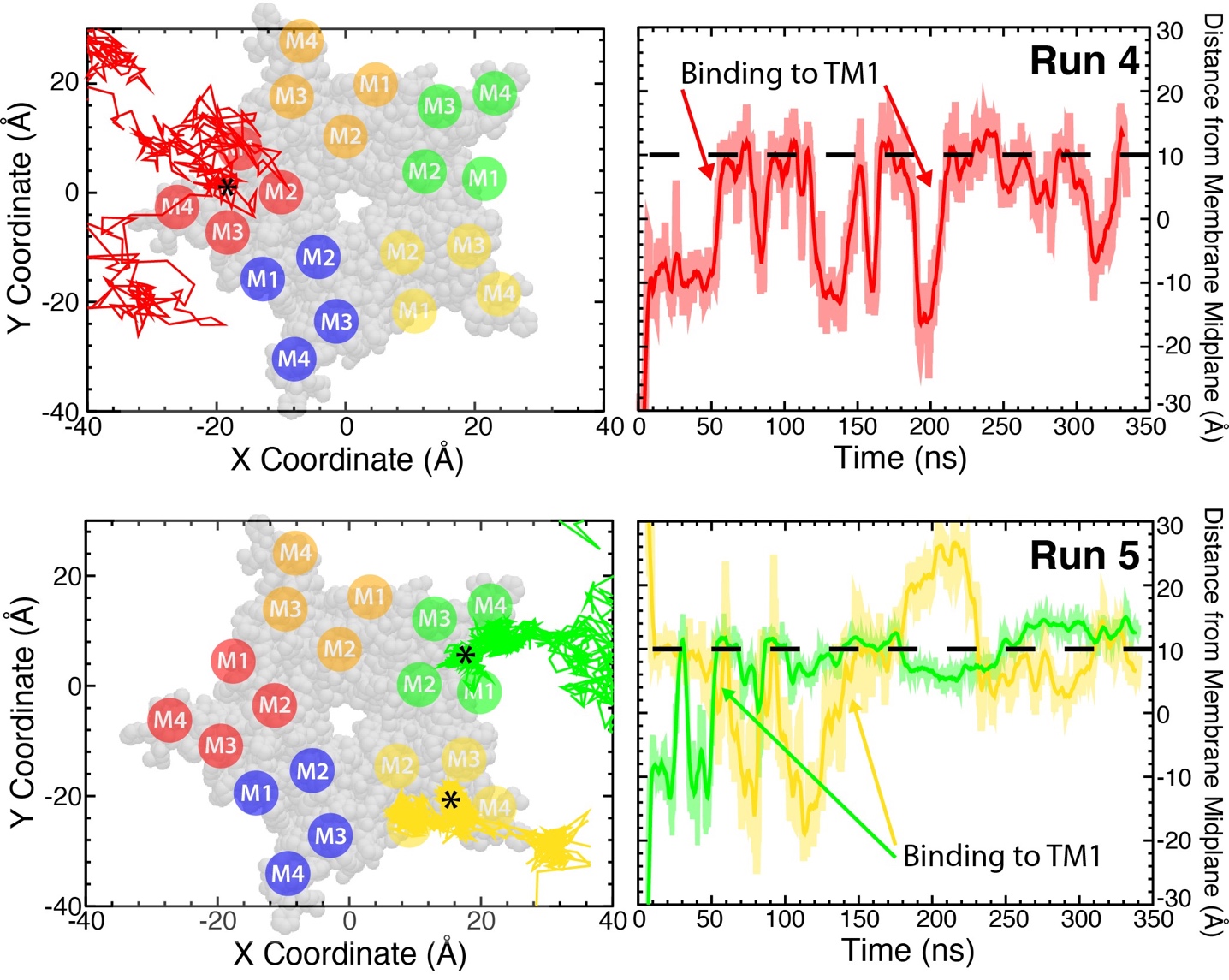


**Figure S2: Binding Pathway of Desflurane to TM1 Site.** Plots of both the *xy*(left column) and *z*-coordinates (right column) of each desflurane molecule judged to be bound is presented for Runs 1–5. (Left) The position of all desflurane molecules bound to the TM1 site for over 50 ns are projected onto the *xy*-plane as a function of time. The color of each trace represents the subunit that each desflurane eventually interacts with. The TMD of GLIC is shown as a transparent gray van der Waals representation with the approximate location of the M1-M4 helices of each subunit shown as circles. The asterisk denotes the position utilized as the TM1 binding site, which was taken from the crystal structure. (Right) Plot of the distance from the membrane midplane as a function of time for each anesthetic molecule. Here, a running average utilizing 5 ns windows is shown as the solid trace and the raw data (every 0.2 ns) is shown as the transparent trace. The dashed line represents the average position of the TM1 site.


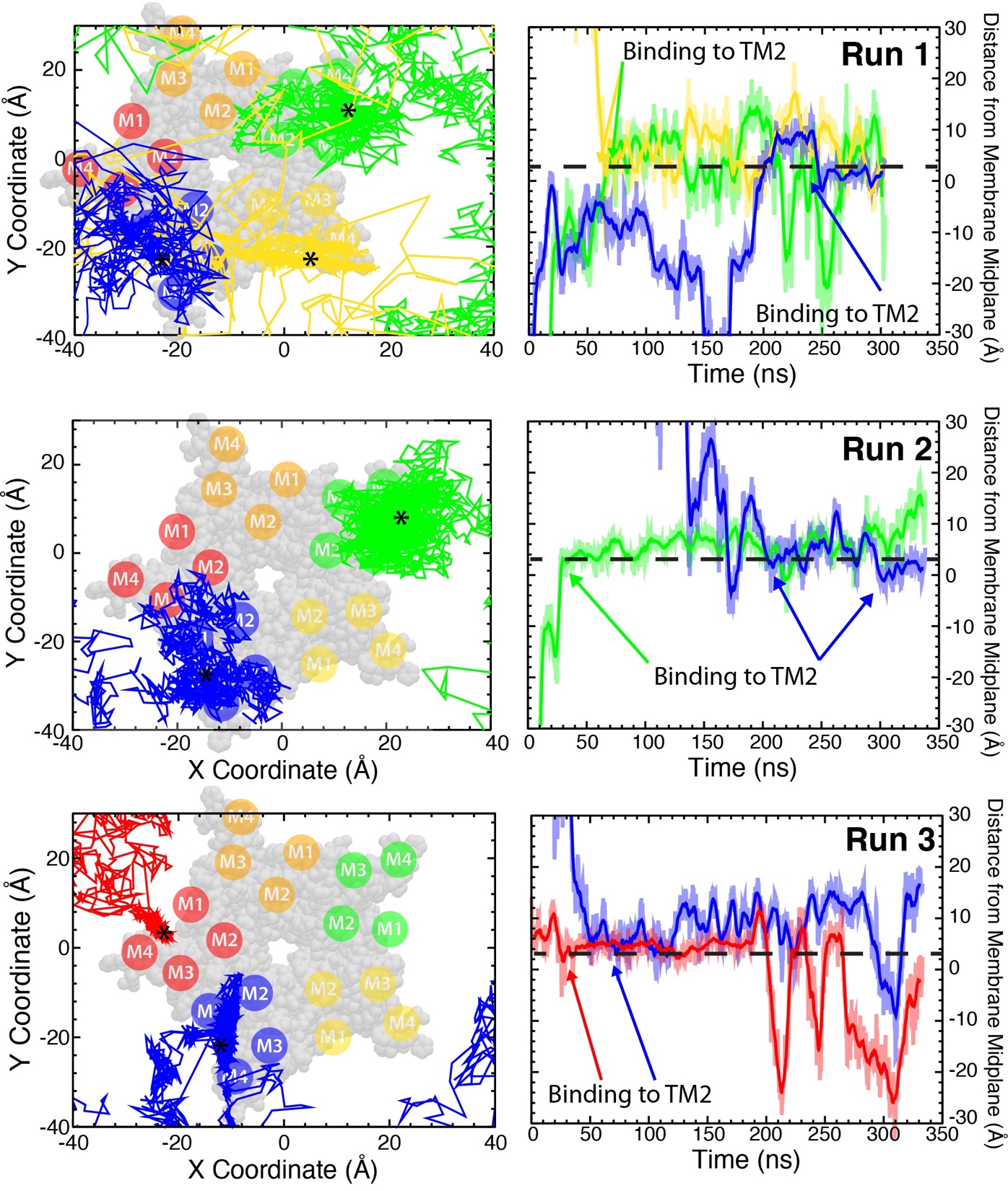


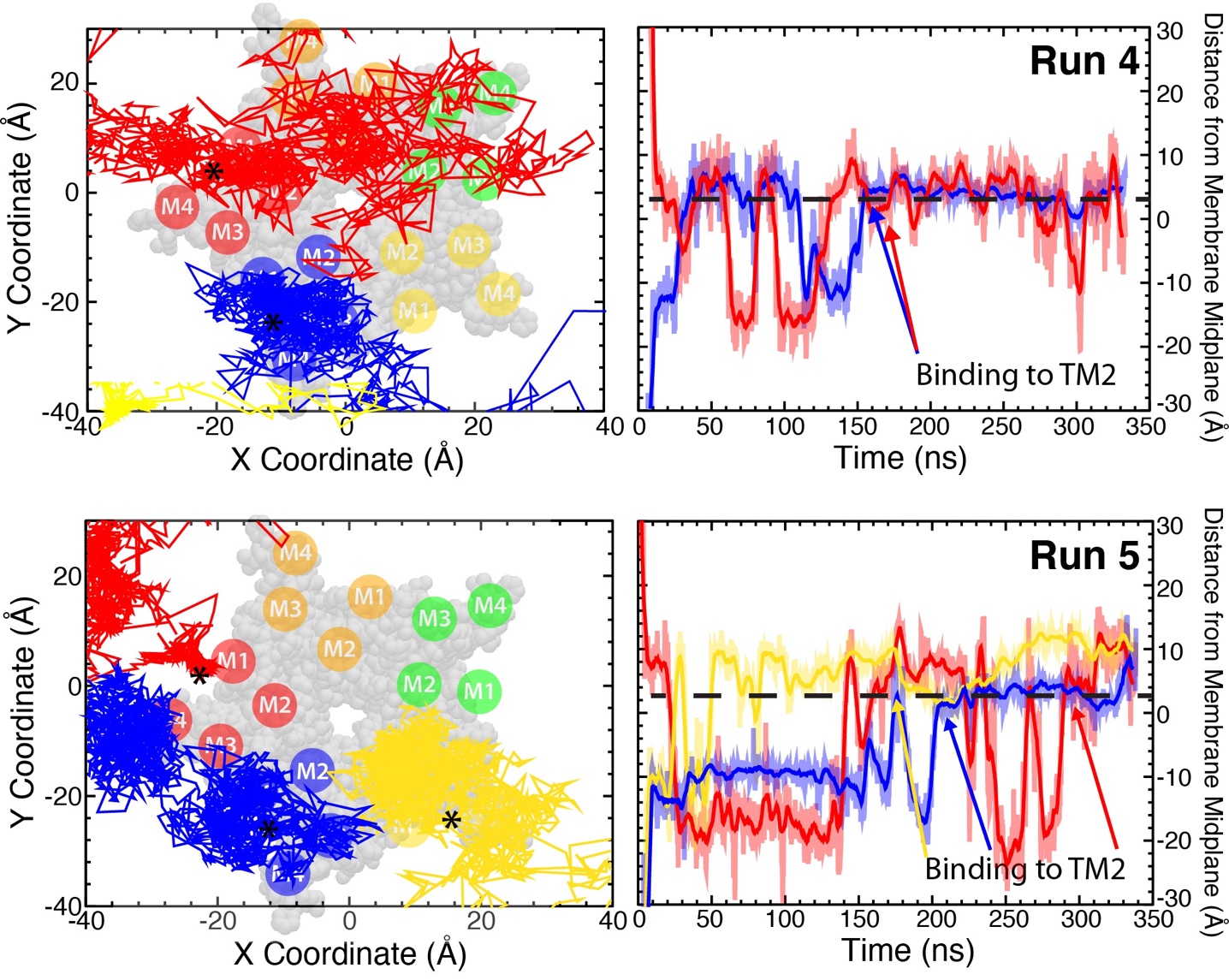


**Figure S3: Binding Pathway of Desflurane to TM2 Site.** Plots of both the *xy*(left column) and *z*-coordinates (right column) of each desflurane molecule judged to be bound is presented for Runs 1–5. (Left) The position of all desflurane molecules bound to the TM2 site for over 50 ns are projected onto the *xy*-plane as a function of time. The color of each trace represents the subunit that each desflurane eventually interacts with. The TMD of GLIC is shown as a transparent gray van der Waals representation with the approximate location of the M1-M4 helices of each subunit shown as circles. The asterisk denotes the position utilized as the TM2 binding site, which was taken from the simulations presented herein. (Right) Plot of the distance from the membrane midplane as a function of time for each anesthetic molecule. Here, a running average utilizing 5 ns windows is shown as the solid trace and the raw data (every 0.2 ns) is shown as the transparent trace. The dashed line represents the average position of the TM2 site.
